# Streamlined Membrane Proteome Preparation for Shotgun Proteomics Analysis with Triton X-100 Cloud Point Extraction and Nanodiamond Solid Phase Extraction

**DOI:** 10.3390/ma9050385

**Published:** 2016-05-18

**Authors:** Minh D. Pham, Ting-Chun Wen, Hung-Cheng Li, Pei-Hsuan Hsieh, Yet-Ran Chen, Huan-Cheng Chang, Chau-Chung Han

**Affiliations:** 1 Institute of Atomic and Molecular Sciences, Academia Sinica, Taipei 10617, Taiwan; minhphd@gmail.com (M.D.P.); amy3_wen@hotmail.com (T.-C.W.); hli@gate.sinica.edu.tw (H.-C.L.); 2 Institute of Biotechnology, Vietnam Academy of Science & Technology, Hanoi 10600, Vietnam; 3 Agricultural Biotechnology Research Center, Academia Sinica, Taipei 11529, Taiwan; pation35@gmail.com (P.-H.H.); yetran@gate.sinica.edu.tw (Y.-R.C.)

**Keywords:** nanodiamond, diamond-enhanced proteolytic digestion, membrane proteomics, mass spectrometry, salt-induced phase separation, cloud point extraction

## Abstract

While mass spectrometry (MS) plays a key role in proteomics research, characterization of membrane proteins (MP) by MS has been a challenging task because of the presence of a host of interfering chemicals in the hydrophobic protein extraction process, and the low protease digestion efficiency. We report a sample preparation protocol, two-phase separation with Triton X-100, induced by NaCl, with coomassie blue added for visualizing the detergent-rich phase, which streamlines MP preparation for SDS-PAGE analysis of intact MP and shot-gun proteomic analyses. MP solubilized in the detergent-rich milieu were then sequentially extracted and fractionated by surface-oxidized nanodiamond (ND) at three pHs. The high MP affinity of ND enabled extensive washes for removal of salts, detergents, lipids, and other impurities to ensure uncompromised ensuing purposes, notably enhanced proteolytic digestion and down-stream mass spectrometric (MS) analyses. Starting with a typical membranous cellular lysate fraction harvested with centrifugation/ultracentrifugation, MP purities of 70%, based on number (not weight) of proteins identified by MS, was achieved; the weight-based purity can be expected to be much higher.

## 1. Introduction

Membrane proteins (MP) are important drug targets and play important roles in enzymatic process, cell adhesion, and transmitting molecular and electrical signals across the cellular envelope. An estimated 26% of human protein-coding genes have been reported to code for membrane proteins [1] and they are major drug targets of modern medicine. Tiefenauer *et al.* recently reviewed the challenges of membrane protein functional assay [2]. Despite their importance, their characterization has been a challenge due to their intrinsic hydrophobicity, low abundance in cells, and large variation in expression level on cellular membranes.

For membranous cellular sample preparation, differential centrifugation is a commonly used first step for fractionating cell lysates into soluble (cytosolic) and insoluble (organelles and membranous) fractions. This method is simple and routinely used, but a significant amount of cytosolic proteins can co-precipitate with membranes. With multiple washes and relevant work up, a typical membrane-enriched sample recovered from ultracentrifugation reaches about 50% in purity assessed by shot-gun proteomics analysis [3]. Extracting the membrane proteome from its nascent lipid bilayer environment typically demands the use of chaotropic chemicals—initially for solubilizing MP and, later on, removal of lipids and purposefully added solubilizing agents turned into a trouble.

Among available MP preparation protocols, phase separation (aka cloud point extraction) is widely employed for isolation and enrichment of MP from a wide range of samples [4,5,6,7] and the MP of interest can be simultaneously concentrated and purified at a large scale. Extraction of MP using phase separation strategies can be traced back to early 1980s. A number of commercial kits based on the phase separation technique are available, albeit many of them were reported with less than satisfactory performance [7]. This gap has to do with the fact that the advertised effectiveness of most commercial kits are based on a small number of selected surrogate proteins that worked well with that particular recipe.

The use of detergents is largely unavoidable for extracting MP from their lipid membranes and removal of high concentrations of detergent to avoid interference with downstream protease digestion and instrumental analysis has been a prime concern for proteome-wide analysis with the powerful and mature mass spectrometry (MS)-based shot-gun proteomics tools. In this work, we set our goal for establishing an in-house routine protocol for membrane proteome preparation aimed for both SDS-PAGE intact MP profiling as well as bottom-up proteomics analysis. The core tool we adopted was derived from our earlier work using surface-oxidize ND for adsorbing soluble proteins from complicated aqueous solutions [8,9,10]. We started with differential centrifugation to first remove cellular nuclei from the lysates and continued with conventional membrane fractions harvested by ultracentrifugation, and further depleted residual cytosolic contaminants with salt-induced phase separation of aqueous Triton X-100 [5]. The solubilized MP in the detergent-rich milieu were then extracted by solid phase extraction (SPE) onto surface-oxidized nanodiamond particles, on which ensuing sample cleanup and enzymatic digestion were conveniently and effectively performed. The Solid Phase Extraction and Elution on Diamond (SPEED) approach had been well-demonstrated for harvesting a wide range of proteins from highly contaminated aqueous environments [11,12,13,14].

Surface-oxidized ND has negative zeta-potential characteristic of cation exchangers at pH above 3 [15] and the surface C-H termination renders ND additional hydrophobic interaction with MP. ND has large surface area, superior inertness, and a density that allows facile formation of aqueous suspension that takes several weeks to settle undisturbed, as well as centrifugal separation and easy handling with common laboratory equipment and manipulations. The advantages of using the Triton detergents include: (i) their wide use in membrane protein sample preparation; (ii) their phase separation property enables easy enrichment and separation of hydrophobic MP from contaminating hydrophilic components abundant in the cytosol; and (iii) their non-ionic nature is more MS-compatible than ionic detergents, e.g., SDS that is extensively used in other sample preparation protocols such as filter-aided sample preparation (FASP) [16] and gel-assisted-digestion [17]. We preferred TX-100 over TX-114 for its better membrane protein extraction efficiency and the convenience of working at ambient temperature throughout sample preparation.

## 2. Results

### 2.1. Two-Phase Membrane Proteome Extraction

While some polymers, ionic liquids, and organic solvents have been demonstrated for solubilizing hydrophobic proteins, detergents remain the most common resort for preparing MP. Aqueous non-ionic detergents are typically mild and exhibit cloud point phenomena that provide convenient means for separation of hydrophobic molecules, enriched in the detergent-rich phase, from hydrophilic components and contaminants. Among the Triton family, TX-114 is widely used for its favorable separation of phases at ambient temperatures, however, we experienced low yields for proteins heavier than 100 KDa that likely represented multi-pass MP using commercial TX-114 two-phase extraction kits. Moreover, spontaneous phase separation at ambient temperatures with aqueous TX-114 entails inconvenience in sample manipulation. To replace TX-114 with TX-100, we reported here a simple but reliable method for inducing two-phase separation in aqueous TX-100 solutions with direct addition of NaCl to induce phase separation and the application of trace amounts of coomassie blue as an indicator for the detergent phase to facilitate the recovery of the separated phases.

**Figure 1:**
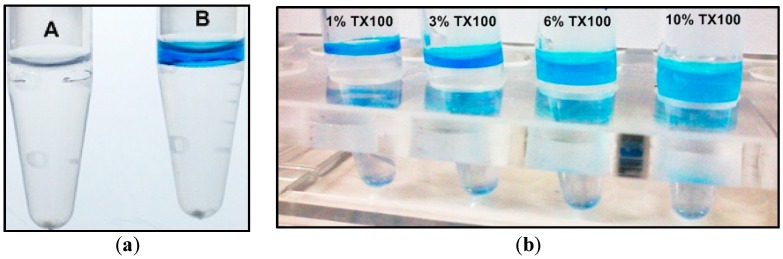
TX-100 two-phase separation performed at ambient temperatures induced by NaCl and coomassie blue added as a phase boundary indicator: (**a**) (A) without and (B) with visualizing dye; and (**b**) stable two-phase separation with increasing amount of TX-100 as marked.

Figure 1 illustrates the effectiveness of using NaCl and coomassie blue for inducing and visualizing the separation of the TX-100 detergent-rich (top layer) and detergent-depleted phases. The left panel consists of two tubes differing only in the addition of 1 µL 0.2% coomassie blue in vial B. The use of visualizing dye was equally helpful with TX-114, except that TX-114 rich phase appears as the bottom layer, and had been applied for detecting residual SDS and TX-114 in protein preparations for MS analysis [18].

The right panel of Figure 1 showed the formation of stable two-phases at different concentrations of TX-100, where the total sample volume was 0.6 mL, and 0.13 g NaCl had been directly added to the sample for inducing phase separation. A seriously conflicting and difficult situation popped up in nearly all MP extraction protocols after MP having been successfully extracted and enriched in the detergent-rich phase in the phase separation manipulation—one needs to deal with potential interference issues arising from the high surfactant contents over the ensuing down-stream purposes. We crudely estimated that the concentration of TX-100 or TX-114 in the detergent-rich phase could reach as high as 30% by weight. Diluting the detergent phase with water or buffer appears to be the simplest means for reducing detergent concentration, however, with concomitant diluting the proteins. Other options include the use of detergent-removal beads, detergent-exchange, dialysis, ion-exchange, and size-exclusion ultracentrifugation or chromatography.

### 2.2. Protein Adsorption by Surface-Oxidized Nanodiamond Particles

As we had hoped at the outset, both liquid phases generated by salt-induced phase separation were compatible with the SPEED platform for facile protein adsorption and contaminant cleanup of MP as described below. Applications of SPEED over water-soluble protein preparation for MS-based proteomics analyses had been well documented in a number of previous publications, where ND displayed superb capability of adsorbing even low concentration peptides/proteins from solutions consisted of high concentrations of detergents and/or salts for SDS-PAGE and MS analysis [11,12,13,14]. In this work, we demonstrated combining this unique property of ND with the newly formulated TX-100 two-phase separation protocol for streamlining the preparation of MP aimed for PAGE and shot-gun proteomics analyses.

**Figure 2:**
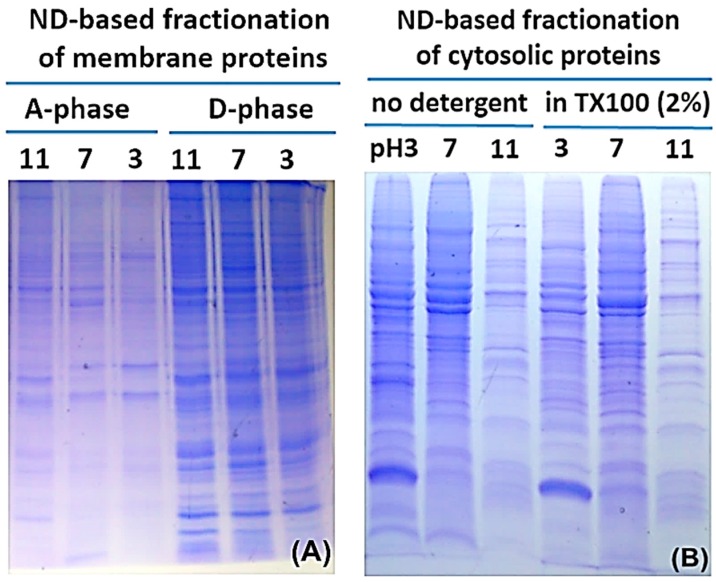
Sequential pH-tuned fractionation and separation of proteins based on SPE with ND: (**A**) fractionation of membrane proteins (MP) enriched sample following cloud point extraction; and (**B**) fractionation of corresponding MP-depleted cytosolic proteins. The input samples used were membranous pellet and cytosolic supernatant, respectively, harvested from ultracentrifugation of mouse myeloma cell lysate. (**A**) For MP, they were first enriched from the membranous pellet with TX-100 two-phase separation and the proteins in the detergent phase (D-phase) are fractionated by sequential pH-tuned extractions with NDs; (**B**) For comparison purposes, the cytosolic supernatant was prepared in two different buffers, with and without the addition of detergents, to test the influence of the surfactant on protein extraction.

**Figure 3:**
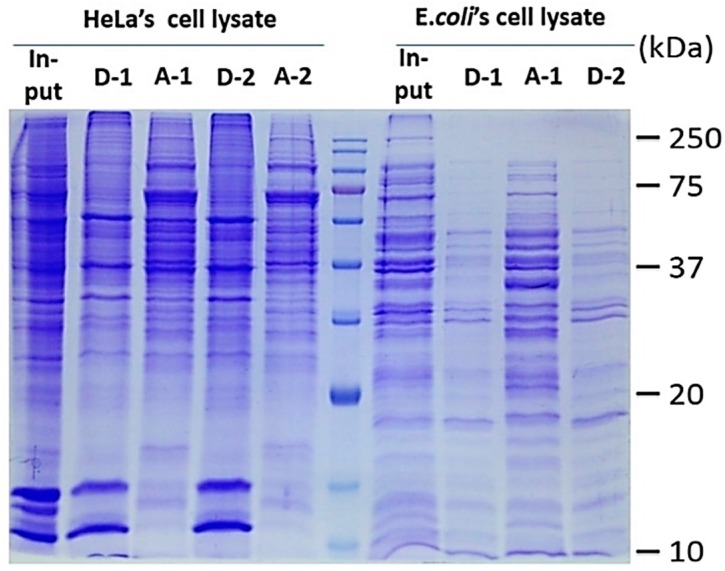
SDS-PAGE analysis of proteins extracted by the SPEED platform following TX-100/H_2_O cloud point extraction from whole cell lysates. D stands for detergent-rich phase, and A for aqueous (detergent-depleted) phase. Lanes marked by “-1” and “-2” represent the absence and presence, respectively, of hydrophobic dye in the phase-separation operation. Input lanes represent the respective whole cell proteome. A-lanes displayed identical patterns as the supernatant from ultracentrifugation. While the PAGE patterns are similar, with or without the aid of phase visualizing dye, recovery of the phases was a much easier task on the operator’s part with its presence.

**Figure 4:**
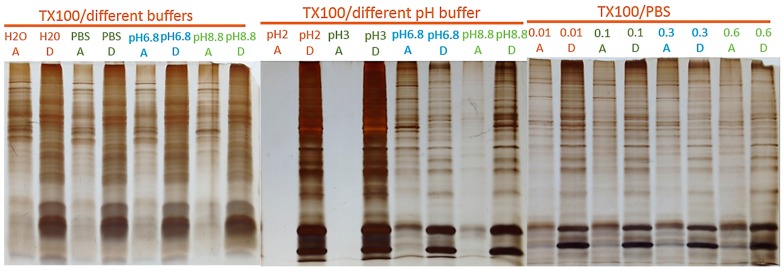
Effects of pH and buffer composition on partitioning of proteins in the detergent and aqueous phases obtained from 4% TX-100/water two-phase separation of a membrane pre-enriched fraction of mouse myeloma cells. A and D stand for aqueous and detergent phases, respectively; pH6.8 and pH8.8 solutions are prepared by Tris buffer (0.5 M) with pH adjusted by HCl; PBS stands for phosphate buffer saline; 0.01, 0.1, 0.3, and 0.6 are the *v*/*v* fraction of 1× PBS in system.

Figure 2, Figure 3 and Figure 4 illustrate the use of ND as the SPE adsorbent in preparation of intact MP from three different types of cells following cloud point extraction. ND was employed to fractionate intact proteins through physisorption at stepwisely reduced pH from both phases, initially pH11, then 7, and lastly pH3. Contaminant (detergent and salts) removal was performed by rinsing protein-laden ND pellet once with 1 mL 0.1% formic acid(FA)/H_2_O 10 volumes of respective pH-adjusted pure water and one final wash with pure water; after each rinsing ND was pelleted by centrifugation (5000× *g*, 3 min). ND-adsorbed protein samples were then ready for SDS-PAGE analysis or proteolysis.

The protocol combining the TX-100 phase separation and the ND protein affinity features was further tested with whole cell lysates from *E. coli* and HeLa cells. Figure 3 shows the SDS-PAGE analyses of proteins extracted by ND from the detergent (D) and aqueous (A) phases of TX-100/H_2_O. Figure 4 further demonstrates ND’s protein adsorption properties over a wide range of solution compositions. These results indicate that ND can extract all proteins, cytosolic and MP alike, effectively from both phases even in the presence of high concentrations of NaCl (up to 2.5 M for soluble proteins from aqueous phase as had been demonstrated [11]) and TX-100 (up to 3% by weight, for hydrophobic proteins from the detergent-rich phase). These results also demonstrated that the SPEED platform made provision for high quality SDS-PAGE analyses with MP conveniently harvested from cloud point extraction. In contrast, direct loading of the samples from either the aqueous phase or the detergent phase, skipping SPEED cleanup procedures, to the SDS-PAGE gels always yielded unresolved protein bands due to excessive salts and detergents. The use of coomassie blue as the detergent phase indicator did not affect the partitioning of proteins in the two phases but its presence greatly facilitated the recovery of the separated phases.

**Figure 5:**
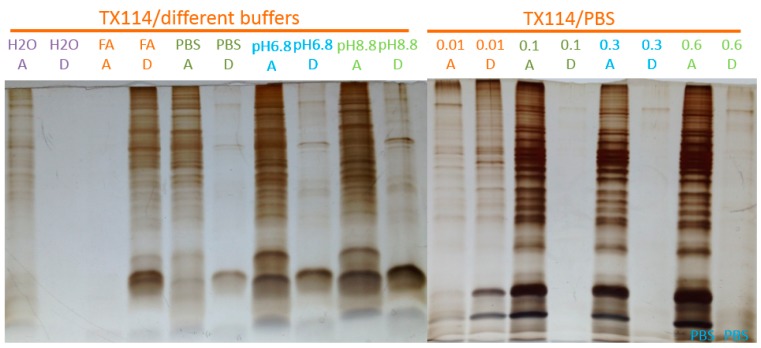
The effects of pH, and buffer composition on the partitioning of proteins in the detergent and aqueous phases obtained from TX-114/water two-phase separation of a membrane pre-enriched fraction of mouse myeloma cells. A and D stand for aqueous and detergent phases, respectively; pH6.8 and pH8.8 solutions are prepared by Tris buffer (0.5M) with pH adjusted by HCl; PBS stands for phosphate buffer saline; 0.01, 0.1, 0.3, and 0.6 are the *v*/*v* fraction of 1× PBS in system.

Figure 4 and Figure 5 show a comparison of cloud point extraction performance using TX-100 *vs.* TX-114, under variable effects of pH, buffer compositions, and ionic strengths on the partitioning of proteins between the two phases for the two detergents. These results indicate that TX-100 is less sensitive toward buffer compositions and ionic strength of buffers used to re-suspend proteins before phase separation than TX-114. However, both Triton detergents were strongly affected by acidic buffers. As a result, SPEED adsorption/fractionation from the detergent-rich phase required careful adjustment of buffer pH. The rule of thumb at work is that low pH favors non-selective protein adsorption.

### 2.3. ND Surface-Enhanced Proteolysis

**Figure 6:**
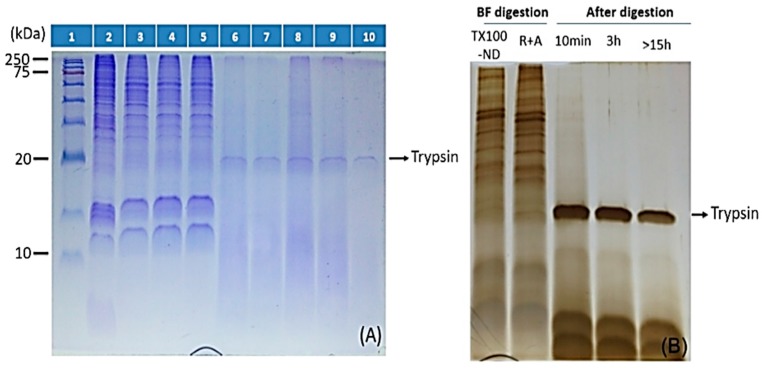
Membrane proteome sample preparation from mammalian cellular membranes by TX-100 salt-induced cloud point extraction coupled with the SPEED platform. Two mammalian cell lines, (**A**) HeLa cells; and (**B**) mouse myeloma cells, were demonstrated for the effectiveness of the method. (**A**) Protein ladders with known molecular weights and a direct input of HeLa cell’s membrane-enriched fraction were loaded in wells 1 and 2, respectively, for reference. Lane 3 presented hydrophobic proteins extracted by SPEED from TX-100 detergent phase; and lane 4 proteins retained after protein reduction and alkylation on ND-surface, but prior to addition of trypsin. Lanes 5–10 monitored the progress of ND-surface enhanced protein digestion: lane 5 overnight without trypsin (a duplicate of lane 4), lanes 6–10 with trypsin/protein ~1/50 and sampled at a digestion time of 10 min, 30 min, 1 h, 3 h, and overnight, respectively; (**B**) BF, TX-100-ND, and R + A stand for before digestion, ND pellet collected in protein extraction from TX-100 detergent-rich phase, and upon completion of reduction/alkylation of proteins on ND-surface, respectively. Where ND was present, proteins/peptides were eluted with 3% SDS loading buffer and only the supernatant was loaded for SDS-PAGE analyses.

Figure 6 examines events occurring on ND surface-protein reduction, alkylation, and tryptic digestion. The difference in lanes 2 and 3 (Figure 6A) originated mainly from only proteins partitioned in the detergent-rich phase was present in lane 3. It is worth noting that even low molecular weight hydrophobic peptides/proteins, as small as 5 kDa, can be effectively captured by ND. The result is consistent with our recent study for bacterial MP using the same ND [12] suggesting broad applicability of ND in concentrating both soluble and MP from a wide range of biological samples. The effectiveness of protein reduction and alkylation of MP were revealed by lanes 3 and 4, and indicated no detectable loss of the proteins. We then examined the time course of tryptic digestion of the proteins (lanes 5–10, Figure 6A, with HeLa cells). As expected, no change occurred overnight when no enzyme was added to the protein-laden ND suspension (lane 5). Results of the time course measurements at 10 min, 30 min, 1 h, 3 h, and overnight, lanes 6–10, respectively, suggested that while digestion was more complete after overnight incubation, most protein bands have largely disappeared after just 10 min of digestion. The enhanced enzymatic activity for specific proteins adsorbed on ND had recently been reported for proteolytic digestion of particulate methane monooxygenase (pMMO), a trimeric bacterial membrane protein [12]. This might be ascribable to an enhancement in protein exposure and denaturation on the ND surface.

The same enhancement in tryptic digestion persisted in treating mouse myeloma cells, as shown in Figure 6B. Taken together, ND-adsorption induced enhancement in tryptic activity had been demonstrated for multiple soluble as well as membrane proteomes, suggesting that the SPEED platform is a universal tool for streamlining proteomics workflow. Note that trypsin was found adsorbed to ND surface, as represented by the intense PAGE band at about 20 kDa. A deeper glimpse of the success of the SPEED platform for membrane proteomics study will be clearer with bottom-up MS analyses of the tryptic peptides as discussed below.

## 3. Discussion

### 3.1. Sequential, pH-Tuned, ND Extraction and Fractionation For “Shot-Gun” Membrane Proteomics

MS is a core analytical tool in contemporary proteomics research, however, membrane protein sample preparation for MS analysis has not been a routine task and generic protocols for this class of important proteins are rare. Removal of purposefully added interfering contaminants, mainly detergents, chaotropes, and salts, prior to MS analysis is of vital importance, but nearly all existing protocols for achieving this goal are time-consuming, labor-intensive, or contribute to significant sample loss. A number of approaches have been developed to improve and facilitate the characterization of MP by MS [19,20,21,22,23,24]. In this section, we focused on the use of the SPEED platform to enrich and prepare membrane protein samples from mammalian cell lines for shot-gun proteomics analyses.

The large majority of researchers who study MP may face the same MS resource constraint like us in relying on predefined analytical conditions set by centralized MS facilities, and we developed this working protocol to serve these researchers. For this purpose, fractionation of proteins was performed to alleviate later excessive dependence on liquid chromatography (LC) separation and extend the membrane proteome coverage by pH-tuned ND extraction in a sequential manner.

We learned from our past experience that protein adsorption of water soluble proteins on ND is pH dependent [12,13]. In this work, we exploited the pH effects on membrane proteome adsorbability on ND with the goal set for improving membrane proteome identification and sequence coverage in shot-gun proteomics analyses. Fractionation of the membrane proteome was achieved through sequential, stepwisely pH-tuned differential adsorption of proteins by ND, first at pH11. As shown in Figure 2A, TX-100/H_2_O extracted phases from denucleated mouse myeloma membranes were each sequentially fractionated into three fractions with the SPEED platform at pH11, pH7, and pH3. Different protein band patterns were obvious in different fractions, especially between the two phases meant to fractionate hydrophilic proteins from hydrophobic ones, indicating the efficacy of the method for membrane protein fractionation. Notably, the SPEED platform tolerated and worked excellently under high concentrations of detergents and salts. Proteins adsorbed on ND allowed facile, yet exhaustive, cleanup of interfering impurities for down-stream analyses, thanks to ND’s density of ~3.5 g/cm^3^ that allowed its easy precipitation by centrifugation. Judging from the tints of the PAGE lanes, >70% of the proteins from the denucleated mouse myeloma membranes partitioned in the detergent-rich phase.

The cytosolic proteome, *i.e.*, supernatant collected from ultracentrifugation of denucleated cell lysate, was also submitted to the pH-tuned protein extraction procedure and analyzed by SDS-PAGE, as shown in Figure 2B. This fractionation approach worked equally well for the hydrophilic proteome, in the presence or absence of 2% TX-100 added for mimicking the detergent-rich phase. These results testified the niche of the SPEED platform following cloud point proteome fractionation.

“Shot-gun” or “bottom-up” membrane proteomics was conducted with standard LC-MS/MS technique to further evaluate the effectiveness of the protocol being developed for fractionating membrane proteomes. Please note that, as the main goal of these MS analyses in this current study was to provide a glimpse of what we actually had harvested from the sample preparation protocol being developed here, only a single LC-MS/MS run was performed with each of the six fractions acquired from one sample manipulation and significant undersampling of the tryptic peptides was anticipated. A typical proteomics-targeted study would have required biological as well as technical replica of (2D-)LC-MS/MS runs that could increase the number of identified proteins by more than 50% from a single run [25].

Proteomics data in Excel file format are provided as supplementary files in nine separate files describing bioinformatics summary of the proteomics findings. With the exception of the final concluding functionality analysis (Figure 9), the bioinformatics criterion we adopted for identification of a protein was that at least two unique tryptic peptides matched *in silico* digestion of the mouse genome. A total of 1630 unique protein families were identified by merging all peptides detected from the six tryptic peptide pools fractionated with SPEED from the mouse myeloma membrane sample, with a false discovery rate of 2% (Table B1 in Excel S1). Peptides in each fraction were analyzed in a single 2 h. LC-MS/MS experiment and the proteins identified in each of the six fractions are presented in Excels S2–S7.

**Figure 7:**
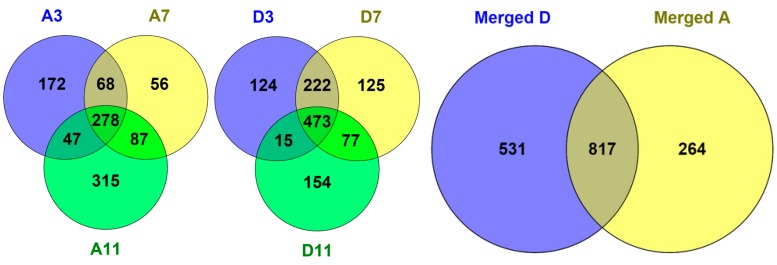
Venn diagrams representing the overlapping of identified proteins between fractions generated by pH-tuned ND fractionation of both aqueous phase (A) and detergent phase (D) at three pH values (pH11, pH7, and pH3).

The partition of identified proteins under the three pH extraction conditions are summarized by the Venn diagram shown in Figure 7, and is in qualitative agreement with the SDS-PAGE protein partition patterns sequentially captured at three distinct pH values (Figure 2A). This Venn diagram was created with Venney 2.1 (BioinfoGP, Spanish National Biotechnology Centre). Note that Mascot identified more proteins from merged peptide pools than does the sum of proteins identified from corresponding component fractions, and this is consistent with general experience. These results demonstrated the effectiveness of this SPEED sample fractionation protocol for preparing membrane proteome for shot-gun protein identification, particularly for researchers who do not have in-house protein fractionation equipment and MS expertise.

Table B8 in Excel S8 summarizes the proteomics findings, 1021 of the 1333 proteins (77%) identified from merging all peptides identified from the three pH fractions derived from the detergent-rich phase were classified as MP by free Internet annotations tools provided by the Gene Ontology Consortium. Further UniProt bioinformatics analysis by “Keywords”, where 1222 cellular component were returned, showed that proteins with the “tag” cytoplasm and membrane yielded 305 (25%) and 752 (62%) (Table B8 in Excel S8), respectively. On the other hand, a similar bioinformatics analysis of proteins identified in the detergent-depleted phase (*i.e*., aqueous phase or A phase) identified 713 MP out of 1066 (67%) annotated proteins, suggesting a 33% non-MP in the A phase. In contrast, 23% of all annotate proteins were non-MP in the D phase, indicating only a weak enhancement in hydrophilic–lipophilic separation was achieved with this current recipe.

**Figure 8:**
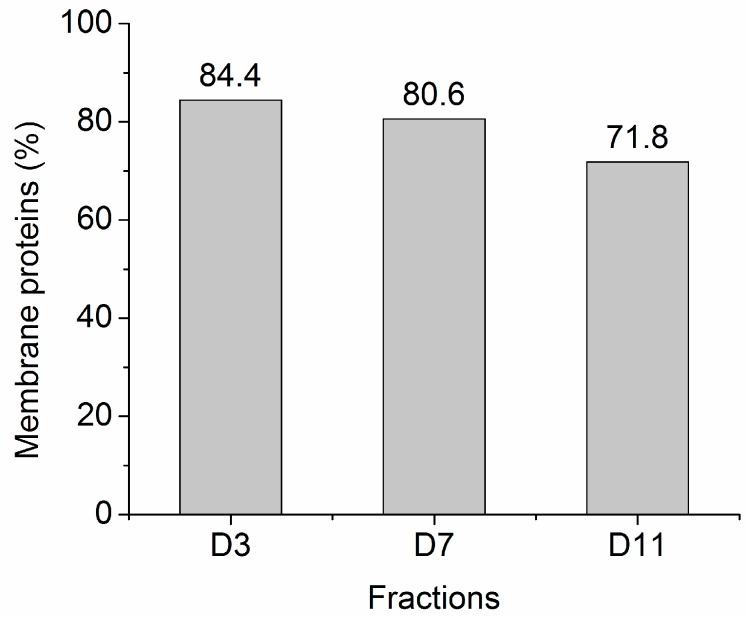
Percentage of membrane proteins identified from three fractions obtained from sequential pH-tuned ND fractionation of the proteins participating in the detergent-rich phase (D-phase). Protein cellular component classification is based on Gene Ontology provided by the UniProt Consortium (EMBL-EBI).

Classification of the identified proteins from sequential pH-tuned ND fractionation of the detergent phase is presented in Figure 8. Most notably, 84.4% and 80.6%, respectively, of the proteins extracted at pH3 and pH7 are classified as MP by Gene Ontology. For comparison, other works on extracting MP using a variety of targeting/labeling strategies involving specific interactions, such as cell surface biotinylation [26], concanavalin A-immobilized magnetic beads [27], detergent-based aqueous two-phase systems [28], or multiple-wash ultracentrifugation [3], typically achieved purities of ~40%–60% in MP.

Table 1 gave additional information on the proteins identified from three pH fractions of the detergent-enriched phase in comparison with proteins participating in the detergent-depleted phase (*i.e*., aqueous phase). Inspection of fractional proteins with transmembrane domain (TM) and the grand average hydropathy (GRAVY) parameters clearly testified the enrichment of highly hydrophobic proteins into the detergent-rich phase. Considering that less than 30% of membrane-originated proteins was partitioned in the detergent-depleted aqueous phase, as reflected in the summed tints of PAGE lanes present in A- *vs.* D-phases (Figure 2A), the actual membrane protein enrichment factor (when protein weight, instead of number identified by MS, is considered) would have been at least a factor of two higher than the data in Table 1 suggest. It is evident that the TX-100/H_2_O binary extraction system effectively enriched MP while depleted cytosolic proteins in the detergent-rich phase.

**Figure 9:**
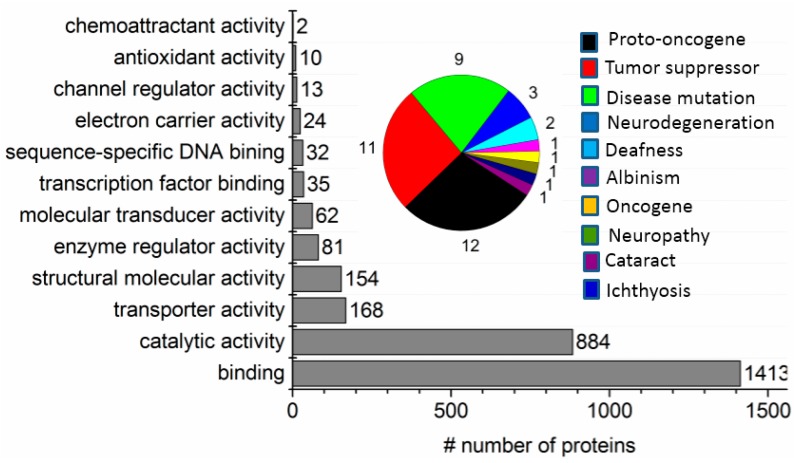
Molecular function classification of all proteins identified with at least one unique matched peptide from six fractions of a whole membrane proteome of cultured mouse myeloma cells fractionated by combination of TX-100 two-phase separation and sequential pH-tuned ND extraction. Pie chart represents proteins associated with biomedical attractions or diseases.

Finally, Figure 9 summarizes the molecular function classification of proteins identified with at least one unique matched peptide from the membrane proteome of cultured mouse myeloma cells.

### 3.2. Considerations in Using this SPEED Protocol for Membrane Protein Samples

We have demonstrated that the ND-based fractionation protocol is effective for separating proteins extracted from denucleated membranes into 6 evenly distributed fractions with enhanced digestive performance that is suitable for outsourced shot-gun MS analyses without developing dedicated HPLC gradients. It should be noted, however, that salt-induced phase separation of non-ionic detergents may not work as straightforwardly as described in this article when a high salt condition is needed at all times for preservation of protein activity during sample purification. In such a case, reducing the operation temperature may stabilize the single phase solution until phase separation is desired. Protein precipitation due to salting out, is another concern in selecting detergents with higher hydrophilicity, which, in turn, usually requires higher salt concentration for phase separation. With TX-100, the detergent-rich phase is the upper layer and precipitated protein pellet, if it occurs, can be conveniently observed.

Lipid rafts, aka detergent-resistant membranes, contains hard-to-reach MP playing key roles in cell signaling and protein sorting, and TX-100 alone at 4 °C is too mild to disrupt such membrane zones. For this purpose higher mass ratio of the detergent over protein and elevated temperatures (≥37 °C) are required to extract and solubilize MP.

### 3.3. Room for Improvement

As an intact protein SDS-PAGE and MS-targeted membrane protein preparation protocol where preservation of protein activity is desirable but not of highest priority, much harsher solubilization conditions that disrupt protein–protein interactions and even denaturing the proteins being extracted can be used. For the low occurrence rate of the amino acids K and R in hydrophobic domains, especially in transmembrane helices, use of only trypsin for digestion could be expected to be less than optimal; alternatively, higher *m*/*z* value for peptide ion detection might worth considering for the longer peptides formed from trypsinolysis. It has not been possible to fractionate a whole-cell proteome into clear-cut membrane/cytosolic fractions due to complicated borderline conditions of the fractionation medium and the intertwined interactions arising from all parts of the protein macro-molecules. Understanding the origin of these seemingly amphiphilic characters in proteins found in both fractions may hold the key to the development of better separation protocols on the proteome level, as well as extracting useful information regarding a particular protein exhibiting such apparent amphiphilicity. Influence of varying detergent/H_2_O ratios, use of different non-ionic detergents or multiple detergent mixtures, and adjustments in ND quantity on the outcome of membrane proteome fractionation are interesting topics for more research.

Proteins of organelle origin, Supplementary data, are present as the simple two-step centrifugation we adopted is known to exclude intact nuclei, but not Golgi apparatus, endoplasmic reticulum, mitochondria, lysosomes and endosomes, from the pelleted membranous fraction that we collectively categorized as membrane proteins. An ideal Venn diagram should have small overlap numbers, which is not the case in Figure 7, and it suggests that, while the MP sample preparation protocol worked, there is much room for further improvement. Figure 7 indicates that a single TX-100/H_2_O phase separation operation we applied resulted in only ~50% differential partition of MS-identified proteins.

## 4. Materials and Methods

Many protocols have been developed for membrane sample preparation, and a large majority started out with a detergent-containing lysis buffer that can interfere with functional group targeted molecular labeling efficiencies of the extracted proteins and we purposefully avoided the incorporation of detergents in the cell lysis protocol for our down-stream 2-phase separation strategy to be deployed without complications. The cell lysis operation we used in this work was adopted from the protocol recommended by the Proteomics Center of University of Nevada, Reno, NV, USA.

Surface-oxidized ND with nominal size of 100 nm was acquired from FND Biotech (ND-COOH, (FND Biotech , Taipei, Taiwan). Pure water was produced with an Elga UHQ water treatment system (ELGA LabWater, Woodridge, IL, USA). All chemicals had been acquired from commercial sources: (1) Thermo Fisher Scientific, all cell-culture agents including Dulbecco’s Modified Eagle Medium (DMEM), fetal bovine serum (FBS), Penicillin Streptomycin (Pen Strep) mixture, Dulbecco’s phosphate-buffered saline (DPBS), and glacial acetic acid; (2) Acros Organics, EDTA, Triton X-100, NaCl, formic acid (FA), Acrylamide/N,N′-Methylenebisacrylamide, ammonium persulfate (APS), tetramethylethylenediamine (TEMED), coomassie blue R250, Na_2_S_2_O_3_, AgNO_3_, formaldehyde, Na_2_CO_3_, and NaHCO_3_; (3) Sigma-Aldrich, glycerol and bromophenol blue; (4) Bio Basic, MgCl_2_; (5) J.T Baker, Tris(base); (6) Riedel-deHaen, HCl; and (7) Burdick & Jackson, methanol.

**Figure 10:**
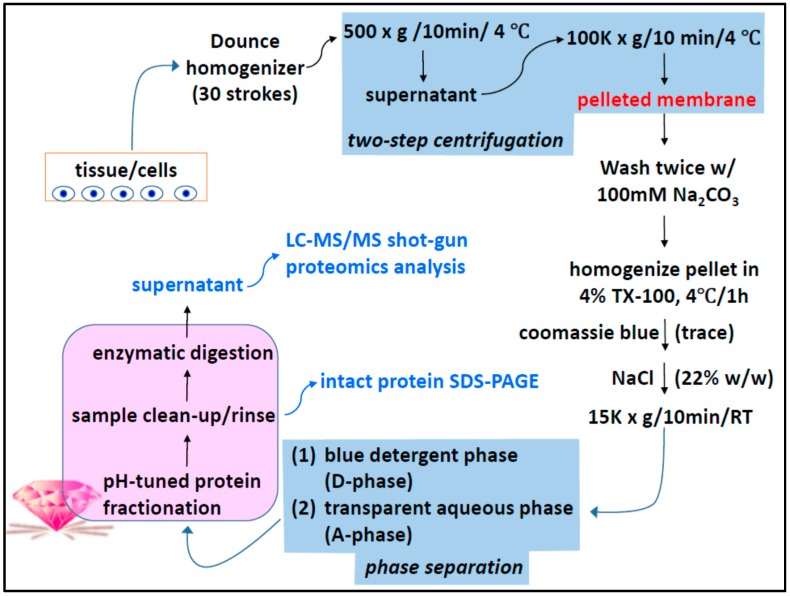
Experimental overview of membrane proteome preparation for MS proteomics identification with the SPEED platform.

An overview of the protocol presented here is shown in Figure 10. Details of long-standing sample manipulation procedures applied in this work, cell culture, preparation of detergent stock solutions, preparation of membrane-enriched fractions by two-step centrifugation, SDS-PAGE, process validation, nano-LC-MS/MS and bioinformatics analysis that did not affect the essence of this presentation are described in the Appendix.

### 4.1. Two-Phase Separation and Enrichment of MP

Two-phase membrane protein extraction with TX-114 has been a common laboratory practice [29] and the details of our manipulation of cultured mammalian cells are described in Appendix. A similar procedure was performed with aqueous TX-100, which has a cloud point of >60 °C for solutions with low ionic strength. To induce phase separation at ambient temperature, 0.22 g NaCl (~3.8 M after dissolution) was directly added to 1 mL of the homogeneous 4% aqueous TX-100-protein mixture, along with 1 µL (0.2%) coomassie blue for later visualization of the detergent phase. Phase separation was similarly assisted by centrifugation, with the TX-100-rich phase containing the visualizing dye floated atop the salty aqueous phase, Figure 1.

### 4.2. MP Fractionation by ND Physisorption from the Detergent-Rich Phase At Stepwisely Decreased pH

Membrane-enriched fraction obtained from two-step centrifugation (refer to Appendix) containing ~100 μg proteins was re-suspended in 1 mL, cold, 4% TX-100/H_2_O using a Dounce homogenizer (30 strokes). The re-suspensions were kept at 4 °C for 1 h to extract and solubilize MP from denucleated cell membrane. This was followed by centrifugation (15,000× *g*, 10 min, 4 °C) to pellet insoluble materials. Then, 0.6 mL of the homogenized supernatant was used for two-phase enrichment of MP using TX-100 as described in the previous two-phase separation section, with 0.13 g NaCl used for inducing phase separation.

The detergent-rich phase obtained from the two-phase separation, ~100 µL, was used for protein fractionation by sequentially and stepwisely reducing the pH for physisorption onto ND. This was done by addition of 50 µL 2 M Na_2_CO_3_ and ~850 µL H_2_O to a final volume of 1 mL. After keeping the suspension at room temperature (rt) for 10 min, the alkalinity was checked with pH indicator paper to ensure pH ~11, and 30 µL of the ND suspension (10 µg/µL in deionized (DI) water) was added to extract proteins. The protein-laden ND was then precipitated by centrifugation (5000× *g*, RT, 3 min); the resulting supernatant was transferred to a new microtube for subsequent protein extractions at reduced pH (pH7 and pH2 in sequence), by adding 6 N HCl. Note that CO_2_ is generated upon acidification and the microtube cap should not be closed immediately. The protein-laden ND pellet was washed twice with 1 mL 0.1% aqueous formic acid (FA) and then 0.5 mL deionized water. The resulting protein-laden ND was either directly used for SDS-PAGE analysis or on-particle digestion for protein identification by shot-gun proteomics. Trials with varying ND/protein (*w*/*w*) having been carried out, the best and reproducible results were obtained with ND/protein (*w*/*w*) falling in the range 10–30.

Proteins partitioned in the detergent-depleted aqueous phase (~500 µL) were also extracted by the same pH-tuned ND extraction procedures as described above for the detergent phase, with the addition of 20 µL TX-100 to adjust the composition to match that of the detergent-rich phase.

### 4.3. Surface-Enhanced Proteolytic Digestion of MP on ND

Digestion of MP adsorbed on the surface of the NDs was adapted from earlier work with some modifications [12]. The protein-loaded ND particles were first washed twice with 1 mL 0.1% FA and once by 0.5 mL H_2_O to remove potential interfering impurities. H_2_O (50 µL) was then added to the sample to re-suspend the protein-binding ND with the assistance of water-bath sonication. Disulfide bonds were reduced by adding 5 µL of 100 mM dithiothreitol (DTT)/100 mM NH_4_HCO_3_ to the suspension followed by incubation at 56 °C for 45 min. A 5 µL aliquot of 100 mM iodoacetamide/25 mM NH_4_HCO_3_ was then added for cysteine alkylation. The mixture was incubated in the dark for 45 min before adding 1 mL 0.1% FA to terminate the reaction. The protein-binding ND was collected by centrifugation (5000× *g*, RT, 3 min.) and washed twice with 0.6 mL of deionized H_2_O to remove residual reducing and alkylating reagents prior to enzymatic digestion.

## 5. Conclusions

A thoroughly tested membrane proteome sample preparation protocol was reported to facilitate researchers who work with membrane proteins and need MS analyses but do not have routine access to complicated LC fractionation platforms. This protocol and its working concept can also work for specific proteomics sample preparation needs with due modifications made to overcome incompatibility with the targeted sample. ND surface enhanced trypsinolysis is particularly useful for bottom-up MP proteomics workflow, as MP have low arginine and lysine contents and their strong hydrophobic nature prevent easy access to digestive enzymes. For research works focusing on digging deeper into membrane proteomics, more rigorous LC-MS/MS proteomics practices that perform replicate runs should be used.

Separation of hydrophilic and lipophilic proteins originating from cellular lysates by detergent/water two-phase extraction was not highly effective and may be improved by using more stringent solvent systems. Nearly all MP purification works we examined reported high-level MP contamination with cytosolic proteins, accounting for typical reported MP purities of 40%–50%. Had this poor hydrophilic–lipophilic protein segregation been an intrinsic nature of protein–protein interactions, MP purification on the proteome level can be ultimately constrained by this very property on the molecular level.

Impressive levels of >80% pure MP was achieved with this SPEED-based cloud point extraction protocol reported here, label-free quantitative proteomics analysis is anticipated to up-adjust these values reported in this work, where purity calculation was based on treating each identified protein as contributing equally in the pool of “purified” proteins. We believe that these contaminating hydrophilic proteins exist in relatively low abundance in the final protein pool.

Finally, we summarized some of the fundamental properties of surface-oxidized ND on which this current SPEED-MP sample preparation protocol was based for enabling interested researchers to better tailor their experiments. Generally speaking, surface-oxidized ND used in this work displays at best weak physicochemical specificity with its overall interaction forces decreasing with increasing pH. Qualitatively, ND has high affinities for most proteins present in a sufficiently complicated protein mixture, such as cytosolic proteomes, serum, or plasma, at low pH, say 3, if free ND surface is available for adsorption. With increasing pH, visually detectable differential affinities, say, monitored by SDS-PAGE, for the protein mixture starts to appear. At high pH, say 8–11, ND’s mitigated ionic property allows weaker interactions with proteins to play a role in selecting its binding partner. This is the reason why fractionation developed in this protocol started with alkaline pH. One should anticipate known irreversible effects of pH and detergents on protein activity and structure for studies related to these fine protein properties.

## Figures and Tables

**Table 1:** Fractional transmembrane domains (TM, obtained with the TMHMM Server, v. 2.0, (Center for Biological Sequence Analysis, Department of Systems Biology, Technical University of Denmark)) and grand average hydrophobicity (GRAVY) of proteins identified from the membrane proteome of cultured mouse myeloma cells fractionated by sequential pH-tuned ND extraction. A-phase presented collective results from the three sequentially pH-tuned extractions with ND.

Origin of MP	TM =1	TM ≥ 2	GRAVY > 0
A-phase	16%	12%	13%
D-pH11	15%	13%	16%
D-pH7	22%	21%	19.2%
D-pH3	23%	26%	24.2%

## Supplementary Materials

The following are available online at www.mdpi.com/1996-1944/9/5/385/s1. Excel S1: Merge of 6 Fractions; Excel S2: Fraction D3; Excel S3: Fraction D7; Excel S4: Fraction D11; Excel S5: Fraction A3; Excel S6: Fraction A7; Excel S7: Fraction A11; Excel S8: Merge of D phase; Excel S9: Merge of A phase.

## Appendix: Supplementary Experimental Information

### A.1. Cell Culture

Human cervical cancer cells (HeLa cells) and mouse myeloma cells were cultured in Dulbecco’s modified Eagle’s medium (DMEM) containing 10% fetal bovine serum (FBS), 100 μg/mL penicillin, and 100 μg/mL streptomycin (Gibco-Invitrogen). Both cell lines were grown at 37 °C and 5% CO_2_ in a humidified air incubator. To harvest the cells, HeLa cells attached to the bottom of the culture plates were first washed twice with 1 × phosphate buffer saline (PBS) and then detached gently with a small plastic scraper. Mouse myeloma cells, on the other hand, were detached with force by pipetting the buffer only, since they were weakly attached to the culture plates. The harvested cells were washed one more time with PBS and stored at −80 °C until use. Cell culture and preparation of the membrane-enriched fractions of *E. coli* cell lysate followed procedures described previously [12].

### A.2. Detergent Stock Solution

Detergent stock solutions (20% (*w*/*w*)) were prepared by mixing 2 g of the respective detergents with 8 gram H_2_O; later use of more diluted detergent solutions were prepared by diluting the stock solution with H_2_O based on volumetric measures.

### A.3. Two-Phase Separation and Enrichment of Membrane Proteins From Intact Mammalian Cells with TX-114

Pellets of approximately 3 × 10^6^ cells (either HeLa or mouse myeloma) were mixed with 1 mL of 2% TX-114/H_2_O buffer and 1 µL 0.2% coomassie blue G250. The mixture was kept at 4 °C for 30–60 min for extracting and solubilizing membrane proteins into micelles. The suspension was then centrifuged (15,000× *g*, 4 °C, 10 min) to exclude insoluble materials. Separation of the bottom TX-114-enriched phase containing the blue indicator from the transparent detergent-depleted phase was facilitated by centrifugation (15,000× *g*, 37 °C, 10 min).

### A.4. Two-Step Centrifugation for Membrane-Enrichment

About 1 × 10^8^ cells (either HeLa cells or mouse myeloma cells) were suspended in 5 mL of cold lysate buffer containing 250 mM sucrose, 1 mM EDTA, and 10 mM Tris-HCl at pH7.4, supplied with protease inhibitor cocktails (Pepstatin, Leupeptin, PMSF). Cells were homogenized with 30 strokes using a Dounce homogenizer. The membrane-enriched fractions were then purified by two-step centrifugation. First, nuclei and unbroken cells were pelleted by centrifugation at 500× *g* for 10 min at 4 °C. Second, the supernatant was ultracentrifuged for 1 h, at 100,000× *g* and 4 °C to pellet the cell membrane. Contamination with cytosolic proteins in the membrane-enriched fraction was removed by washing/ultracentrifugation twice with 100 mM Na_2_CO_3_. Final membranous product was aliquoted and stored at ‒80 °C until use. Protein concentration was measured by BCA protein assay.

Note that unless the nuclei had been removed prior to cloud point membrane protein extraction, exceedingly high concentrations of core histones unavoidably contaminated the detergent-rich phase; as a result, we recommend adopting the conventional two-step centrifugation procedure for membrane proteome preparation of mammalian cells.

### A.5. Sodium Dodecyl Sulfate Polyacrylamide Gel Electrophoresis (12% SDS-PAGE)

Protein-binding ND was first washed with 0.1% FA and then deionized water to remove loosely bound moieties such as salts and detergent before elution with 30 µL SDS-PAGE loading buffer (3% SDS, 1.5% DTT, 0.1% bromophenol blue, 0.5 M Tris-HCl pH6.8, 10% glycerol, 95 °C, 5 min). Only the supernatants containing solubilized proteins were loaded onto the gel for analysis.

### A.6. Process Validation

In the early stage of method development, SDS-PAGE was used for quick, in-house, evaluation of the completeness of digestion at given time elapses. The protein-binding ND samples were divided evenly into four parts. One part was saved as a qualitative reference for starting sample before digestion, the other three parts were used for ND-surface enhanced digestion by adding 200 µL of 50 mM NH_4_HCO_3_ containing trypsin (sequencing grade, Promega, Madison, WI, USA; protein/trypsin 20/1) followed by incubation at 37 °C for 10 min, 3 h., and 15 h., respectively, and the enzymatic activity was quenched at the targeted time by adding FA to 0.1% final concentration. Small tryptic peptides spontaneously desorbed from ND at the alkaline pH of digestion buffers (50 mM NH_4_HCO_3_) and were separated from the nanoparticles by centrifugation (5000× *g*, 3 min, RT). Residual, loosely bound tryptic peptides was recovered by washing the nanoparticles with 200 μL of 50 mM NH_4_HCO_3_ and then 200 µL of 100% ACN. The peptide supernatants for each individual pH condition were pooled and dried with a vacuum centrifugal device. The undigested protein-laden ND that had been set aside for presenting the starting proteome, and the dried tryptic peptides were re-suspended with PAGE loading buffer and analyzed by SDS-PAGE, and was utilized to quickly monitor the completeness of digestion.

For shot-gun proteomics analysis, the entirety of protein-binding ND samples for each individual pH conditions were used for ND-surface enhanced digestion as described above. The dried tryptic peptides were re-suspended with 0.1% aqueous FA and desalted by Ziptip C18 (Millipore) in compliance with the requirements of the Common Mass Spectrometry Facilities in Academia Sinica (Taiwan) for protein identification by LC-MS/MS.

### A.7. Nano-LC-MS/MS and Bioinformatics Analysis

A reversed-phase nanoLC (Waters nanoAcquity, Milford, MA, USA) coupled to LTQ-Orbitrap XL hybrid mass spectrometer (Thermo Fisher Scientific, Waltham, MA, USA) operated by a specialist at the MS core facility was used for protein identification (ID) of peptides generated from ND’s surface-enhanced digestion of membrane proteins. About 0.5 µg peptide mixtures were loaded onto a 75-µm ID, 25-cm length C18 BEH column (Waters) packed with 1.7-µm particles with a pore size of 130 Å, and were separated using a segmented gradient in 120 min from 5% to 40% solvent B (0.1% FA in ACN) at a flow rate of 300 nL/min and a column temperature of 35 °C. The mass spectrometer was operated in the data-dependent mode and with real time recalibration on the lock mass. Briefly, survey, full-scan, MS spectra were acquired in the orbitrap (*m*/*z* 350–2000) with the resolution set to 60,000 at *m*/*z* 400 and automatic gain control (AGC) target at 10^6^. The 10 most intense ions were sequentially isolated for CID MS/MS fragmentation and detection in the linear ion trap (AGC target at 7000) with previously selected ions dynamically excluded for 90 s.

Raw files were transformed to Windows Installer Merge Module (msm) files by RAW2MSM (v. 1.10, Matrix Science Inc., Boston, MA, USA) software using default parameters and without any filtering, charge state deconvolution, nor de-isotoping. The msm files were searched against the Sprot database, using a Mascot Daemon 2.4.0 server (Matrix Science Inc., Boston, MA, USA). Search criteria used were trypsin digestion, variable modifications set as carbamidomethyl (C) and oxidation (M), allowing up to two missed cleavages, mass accuracy of 10 ppm on the parent ion and 0.60 Da on the fragment ions.

The false discovery rate in protein identification was evaluated by searching against a randomized decoy database created by MASCOT using identical search parameters and validation criteria. Protein subcellular localization, biological process, and molecular functions were obtained from UniProtKB tool (UniProt Consortium (EMBL-EBI)). The number of transmembrane helices (TM) in each of the annotated proteins was predicted by TMHMM 2.0 (Center for Biological Sequence Analysis, Department of Systems Biology, Technical University of Denmark). Protein GRAVY scores were determined using the free Internet tool GRAVY Calculator (University of Greifswald, Greifswald, Germany). Proteins annotated with the term “membrane” by GO annotation, or with TM ≥1, or positive GRAVY scores, were classified as membrane proteins.
